# Age/autoimmunity-associated B cells in inflammatory arthritis: An emerging therapeutic target

**DOI:** 10.3389/fimmu.2023.1103307

**Published:** 2023-01-24

**Authors:** Zhen-yu Li, Ming-Long Cai, Yi Qin, Zhu Chen

**Affiliations:** Department of Rheumatology and Immunology, the First Affiliated Hospital of University of Science and Technology of China (USTC), Division of Life Sciences and Medicine, University of Science and Technology of China, Hefei, China

**Keywords:** age-associated B cells, inflammatory arthritis, autoimmune diseases, immunopathogenesis, therapeutic target

## Abstract

Age/autoimmunity-associated B cells (ABCs) are a novel B cell subpopulation with a unique transcriptional signature and cell surface phenotype. They are not sensitive to BCR but rely on TLR7 or TLR9 in the context of T cell-derived cytokines for the differentiation. It has been established that aberrant expansion of ABCs is linked to the pathogenesis of systemic autoimmune diseases such as systemic lupus erythematosus. Recently, we and other groups have shown that increased ABCs is associated with rheumatoid arthritis (RA) disease activity and have demonstrated their pathogenic role in RA, indicating that targeting specific B cell subsets is a promising strategy for the treatment of inflammatory arthritis. In this review, we summarize the current knowledge of ABCs, focusing on their emerging role in the pathogenesis of inflammatory arthritis. A deep understanding of the biology of ABCs in the context of inflammatory settings *in vivo* will ultimately contribute to the development of novel targeted therapies for the treatment of inflammatory arthritis.

## 1 Introduction

Inflammatory arthritis describes a group of chronic systemic diseases that may affect joints as well as other organs in the body, leading to disability and organ damage that significantly reduce the quality of life (1). These include rheumatoid arthritis (RA), juvenile idiopathic arthritis (JIA), ankylosing spondylitis (AS), psoriatic arthritis (PsA) and other spondyloarthritis. Although the pathogenesis remains incompletely understood, many types of inflammatory arthritis share certain autoimmune features, where genetic and environmental factors operate to activate an adaptive immune response and elicit autoantibody production (2). In addition, the interactions between synovial-like fibroblasts (FLS), macrophages, and infiltrated lymphocytes induce the production of a variety of inflammatory cytokines, which perpetuate the synovial inflammation development and ultimately result in cartilage and bone destruction in RA (3, 4).

B cells have long been recognized as a central player in adaptive immunity (5). The roles of B cells in autoimmune diseases include cytokine secretion, antigen presentation and autoantibody production (6–8). Despite the well-known association with autoantibodies, knowledge of the role of B cells in the pathogenesis of synovitis is limited (9). Indeed, pathogenic B cell subsets have been found to accumulate in the synovial tissue and synovial fluid (SF) of RA patients which correlated with disease activity and joint damage (10, 11). Furthermore, the efficacy of the B cell depleting anti-CD20 monoclonal antibody in the treatment of active RA has been demonstrated, highlighting a crucial contribution of B cells in the pathogenesis of RA (12). Notably, the underlying mechanisms of disease amelioration following B cell depletion have not been fully elucidated since plasma cells are not affected during treatment (12). Considering that depletion of whole B cells raises the risk of severe infections (13) and that B cells are a highly heterogeneous population, it is conceivable that targeting specific pathogenic B cell subsets rather than pan-B cells would be a more promising strategy for the management of inflammatory arthritis such as RA.

Age/autoimmunity-associated B cells (ABCs) are a novel B cell subpopulation that was first identified to accumulate in the spleen of aged female mice as well as in autoimmune-prone mice (14–16). In contrast to follicular B (FoB) and marginal zone B (MZB) cells, ABCs are characterized by the expression of myeloid markers and do not divide in response to B-cell receptor (BCR) signaling (14, 15). Importantly, ABCs were shown to secrete autoantibodies upon stimulation *in vitro*, and depletion of these cells *in vivo* results in a reduction of autoantibody levels, implicating their pathogenic role in the development of autoimmunity (15). In addition, ABC-like cells are also elevated in various infectious diseases such as influenza and malaria (17–20). The observation that ABCs increased in human systemic autoimmune diseases including systemic lupus erythematosus (SLE), scleroderma, Sjögren’s syndrome (SS) and multiple sclerosis multiple sclerosis (MS) further established a link between ABCs and autoimmune diseases (15, 21–26). In addition, researchers have found the expansion of CD21^low^ B cells, a part of ABCs, in immune deficiency (27, 28). Recently, by combining mass cytometry and single-cell RNA sequencing analyses, Zhang et al (29) revealed that ABCs were expanded in the synovium of RA patients, suggesting a potential role of ABCs in inflammatory arthritis. Consistently, a recent work in our lab has confirmed the expansion of ABCs both in the peripheral blood and in the synovial tissue of RA patients (30). Our findings demonstrated a distinct transcriptomic feature of RA ABCs, which may impact their ability to migrate into the inflammatory joints, where they induce FLS to an aggressive phenotype and perpetuate synovitis development (30).

Herein, we summarize the current knowledge of ABCs, focusing on their emerging role in inflammatory arthritis. ABCs formation, especially in the context of inflammatory settings *in vivo*, are discussed. By better understanding the biology and function of ABCs in arthritis, novel insights into the pathogenesis of disease and targeting strategies of ABCs for the treatment of inflammatory arthritis are anticipated.

## 2 Diverse nomenclatures of ABCs

Hao et al. and Rubtsov et al. first described a distinct murine B cell subset that accumulates with age in the spleen and is thus termed age-associated B cells (14, 15). Rubtsov et al. found that this unusual population also appears in young lupus-prone mice and in the blood of patients with several autoimmune diseases (15). Importantly, these cells secrete autoantibodies upon stimulation *in vitro*, and depletion *in vivo* led to a reduction in autoantibodies, suggesting that ABCs might have a potential role in the development of autoimmunity (15). Although the exact markers used to define ABCs differ between these two reports, they shared several key features such as being refractory to BCR and CD40 stimulation whereas responsive to innate stimuli such as Toll-like receptor (TLR) 7 (14, 15).

Since these early observations, the expression of the transcription factor T-bet, which has long been associated with lineage specification of Th1 cells, has become a well-known marker and regulator of ABCs (31). Hence, they are mentioned as T-bet^+^ B cells or CD11c^+^T-bet^+^ B cells in various studies (17–19, 21, 22, 32–36). Other B cell subsets that share at least some ABCs characteristics based on phenotypic and transcriptomic analyses, have also been reported (23, 24, 37–47). For example, aberrant expansion of B cells that lack IgD, CD27 and CXCR5 (DN2 B cells) has been described in SLE patients and shown to be correlated with disease activity and clinical manifestations (23). Interestingly, DN2 B cells shared phenotypic and functional features with activated naive B cells (aNAV), a subset that they reported earlier in SLE patients (23, 41).

It is now generally considered that ABCs are a heterogeneous population, which might partly account for the lack of a uniform definition and the various phenotyping criteria applied among different groups. Despite the diverse nomenclature that has been used to describe these cells (**Table 1**), ABCs are actually emerging as key players in many pathophysiological settings, raising the possibility that this compartment may represent a common pathway in autoimmune-mediated disorders. While we recognize that the term “ABCs” may not an ideal terminology since they are not associated with age in many disease settings including SLE and RA (22, 30), we adopt this nomenclature in our review to encompass ABCs and ABC-like populations. This is in line with most recent comprehensive reviews on the subject that focus on different aspects (31, 57–59).

**Table 1:** Diverse nomenclatures used to describe ABCs and ABC-like populations.

Species	Nomenclatures	Model	Cell markers	Reference
Mouse	Age-associated B cells	Old mice	CD19^+^, CD21/35^-^, CD23^-^, CD43^-^, AA4.1^-^	(14)
		Old mice, lupus-like autoimmune disease	B220^+^, CD19^+^, IgM^+^, CD11b^+^, CD11c^+^	(15)
		Collagen-induced arthritis	B220^+^, CD11c^+^, T-bet^+^	(30)
		Lupus	B220^+^, CD19^+^, CD93^-^, CD43^-^, CD21^-^, CD23^-^, CD11c^+^, T-bet^+^	(33)
		Lupus	B220^+^, CD19^+^, CD11b^+^, CD11c^+^, T-bet^+^	(48)
		Lupus	B220^+^, CD19^+^, CD11b^+^, CD11c^+^	(49)
		Influenza virus infection	Fas^+^GL7^-^, CD11b^+^, CD11c^+^, T-bet^+^	(20)
	Age-associated/Autoimmune B-Cell	Lupus	B220^+^, CD11c^+^, T-bet^+^	(50)
	CD11c^+^T-bet^+^ B cells	Acute lymphocytic choriomeningitis virus infection	B220^+^, CD19^+^, CD44^high^, CD11c^+^, T-bet^+^	(51)
		Chronic graft-versus-host disease lupus	CD19^+^, CXCR3^+^, CD11c^+^, T-bet^+^	(34)
	Age-associated/Autoimmune B-Cell	Lupus	CD19^+^, CD11c^+^, T-bet^+^	(52)
	T-bet^+^ B cells	Obesity	CD19^+^, CD21^-^, CD23^-^, CD69^+^, Nur77^+^ CD11c^+^, T-bet^+^	(35)
	T-bet^+^ memory B cells	Influenza virus infection	CD19^+^, B220^+^, IgD^-^, T-bet^+^	(42)
		Ehrlichia muris infection	CD19^+^, CD80^+^, PD-L2^+^, T-bet^+^	(43)
	Atypical memory B cells (atMBCs)	Plasmodium infection	CD19^+^, CD21^-^, CD27^-^, FCRL5^+^, CD86^+^, CD40^+^, CD11b^+^, CD11c^+^, T-bet^+^	(44)
		Plasmodium infection	CD19^+^, $MSPl_{21}^{+}$ , FCRL5^+^, CD11b^+^, CD11c^+^	(45)
Human	Age-associated B cells	Rheumatoid arthritis, systemic sclerosis, systemic lupus erythematosus	IgD^-^, IgM^-^, IgG^+^, CD38^low^, CD5^high^, CD80^high^, CD86^high^, CD20^high^, CD23^-^, CD27^high^	(15)
		Rheumatoid arthritis	CD19^+^, CD27^-^, IgD^-^, CD21^-^, CD11c^+^	(30)
	CD11c^+^T-bet^+^ B cells	Systemic lupus erythematosus	CD19^+^, CD11c^+^, T-bet^+^	(22)
	Double-negative (DN2) B cells	Juvenile Idiopathic Arthritis	CD19^+^, CD21^low/-^, CD27^-^, IgM^-^, CD11c^+^	(53)
		Obesity	CD19^+^, IgD^-^, CD27^-^, CD21^low^, CD95^+^, CD11c^+^, CD86^+^, HLADR^+^, PD1^+^, T-bet^+^	(46)
		Systemic Lupus Erythematosus	CD19^+^, IgD^-^, CD27^-^, CD11c^+^, CXCR5^-^	(54)
		Systemic Lupus Erythematosus	CD19^+^, IgD^-^, CD27^-^, CD11c^+^, CXCR5^-^, T-bet^+^	(55)
		Systemic Lupus Erythematosus	CD19^+^, IgD^-^, CD27^-^, CD21^-^, CD11c^+^, CXCR5^-^	(23)
	T-bet^+^ B cells	Obesity	CD19^+^, CD11c^+^, T-bet^+^, CD69^+^	(35)
		HIV infection	CD19^+^, CD27^-^, CD21^-^, T-bet^+^	(36)
		Systemic lupus erythematosus, rheumatoid arthritis, HIV infection	CD19^+^, CD21^low^, T-bet^high^	(56)
	T-bet^+^ memory B cells	HIV infection	CD19^high^, CXCR3^+^, CD20^+^, CD11c^+^, T-bet^high^	(47)
		Influenza virus infection	CD19^+^, CD21^-^, IgD^-^, T-bet^+^	(42)
	atMBCs	Malaria exposed	CD19^+^, FCRL5^+^, CXCR3^+^, CD95^+^, CD11c^+^, T-bet^+^	(19)
	Age-associated-like B cells	Systemic lupus erythematosus	CD19^+^, IgD^-^, CD27^-^, CD21^-^, CD11c^+^	(54)
		Granulomatous lung diseases	CD19^+^, CD21^low^, FcRL 2-5^+^, CD11c^+^	(26)

## 3 Origin and differentiation of ABCs

### 3.1 Origin of ABCs *in vivo*

Since discovery, interest in the origin and generation routes of ABCs has been growing. Early studies in a murine system indicated that ABCs can be generated from FoB cells under appropriate conditions rather than from B cell senescence (14). More recent studies revealed that ABCs display characteristics of antigen-experienced cells and fulfill the criteria for memory B cells (60, 61). Sequence analyses of heavy and light chain genes from ABCs showed that they express a diverse repertoire of V_H_ and Vκ genes with significant somatic hypermutation, implying that ABCs are germinal center (GC) originated and have undergone stimulation from antigens over time (61). Furthermore, these authors found that neither major histocompatibility complex-II (MHC-II)-deficient nor CD40-deficient FoB cells could give rise to ABCs, indicating that cognate T cell help is required for ABCs generation (61). However, conflicting results have been shown that ABCs can be yielded *in vitro* without CD40 ligation (55, 62). Similarly, while ABCs express GC-associated surface markers such as CD95 and Peanut Agglutinin (PNA) in the early phase of immune response, a very recent study in a viral infection model demonstrated that ABCs developed independently of GC formation and exhibited distinct phenotypic and transcriptional profiles from GC B cells (51). As infection resolves, ABCs localize to the marginal zone of the spleen, forming a GC-independent memory subset capable of rapid recall responses and contribute to antibody production (51). These seemingly contradictory results, however, are not mutually exclusive, since ABCs may arise through multiple routes depending on signals and context they encounter *in vivo*.

### 3.2 Innate and adaptive signals in regulating ABCs differentiation

It has been well established in early studies that, BCR ligation, either alone or with CD40/CD40L co-stimulation, are not sufficient for ABCs generation (14, 15). In contrast, the participation of innate sensor signals, particularly the engagement of TLR7 and TLR9, with subsequent exposure to T cell-derived cytokines, are crucial in driving ABCs differentiation *in vitro (* 14, 15). Interestingly, TLR7 locates on the X-chromosome and could partially escape X-chromosome inactivation. The expression of TLR7 is critical in controlling sex-specific differences observed in mice on a lupus-prone background, as a dual TLR7 expression in male lupus mice results in higher ABCs formation and more severe organ damage (48). The requisite for TLR7 as a prime driver of ABCs in autoimmune-prone mice was also observed in patients with SLE (23). In a very recent study, Brown et al. described missense TLR7^Y264H^ variant in a child with severe SLE and confirmed that this variant cause lupus when introduced into mice (63). They further showed that enhanced TLR7 signaling drives aberrant accumulation of ABCs and GC B cells in a cell-intrinsic manner (63). In line with these findings, genetic ablation of either TLR7 or myeloid differentiation factor 88 (MyD88), an adaptor protein downstream of TLR7, results in a lack of ABCs in aged and autoimmune mice (15, 48). Besides, the MyD88 deficient mice showed less T-bet expression in B cells also indicated the important role of MyD88 in the formation of ABCs (64). In contrast to TLR7, the role of TLR9 in ABCs differentiation is still controversial. *In vitro* experiments showed that the cross-action between BCR and TLR9 can synergistically increase T-bet expression in B cells (65). Similarly, TLR9 along with interferon-γ (IFN-γ) receptor are essential signals in promoting the development of ABCs in a malaria infection model (66). However, evidence have been emerging that TLR9 seems to exert a protective role in SLE (67). This might be explained by the capacity of TLR9 in limiting the stimulatory activity of TLR7.

In addition to innate stimuli, ABCs are poised to differentiate under adaptive signals which include BCR ligation and circumscribed cytokine milieu. Among the cytokines, interleukin (IL)-21 and IFN-γ are the most important in promoting ABCs formation whereas IL-4 negatively regulates ABCs fate in the context of IL-21 (49, 56, 62, 68, 69). In addition, our recent work demonstrated that IL-13 receptor α1-mediated signaling regulates ABCs generation and differentiation in lupus-prone mice (50). Interestingly, the combination of IL-21 and IFN-γ stimulation induce the highest ABCs formation both in infection and in lupus murine models (49, 70, 71). The regulatory cytokine interplay between IL-21, IFN-γ and IL-4 was confirmed *in vivo* through experiments in various knockout mice following influenza or *Heligmosomoides polygyrus* challenge, examples of Th1 versus Th2 predominated immune responses, respectively (62). Notably, the effects of IL-21 and IFN-γ in promoting ABCs generation are not identical, since IL-21 promotes the upregulation of both T-bet and CD11c while IFN-γ induces the expression of T-bet but not of CD11c (62), suggesting the differences in the molecular mechanisms employed by these cytokines in regulating ABCs differentiation.

**Figure 1:**
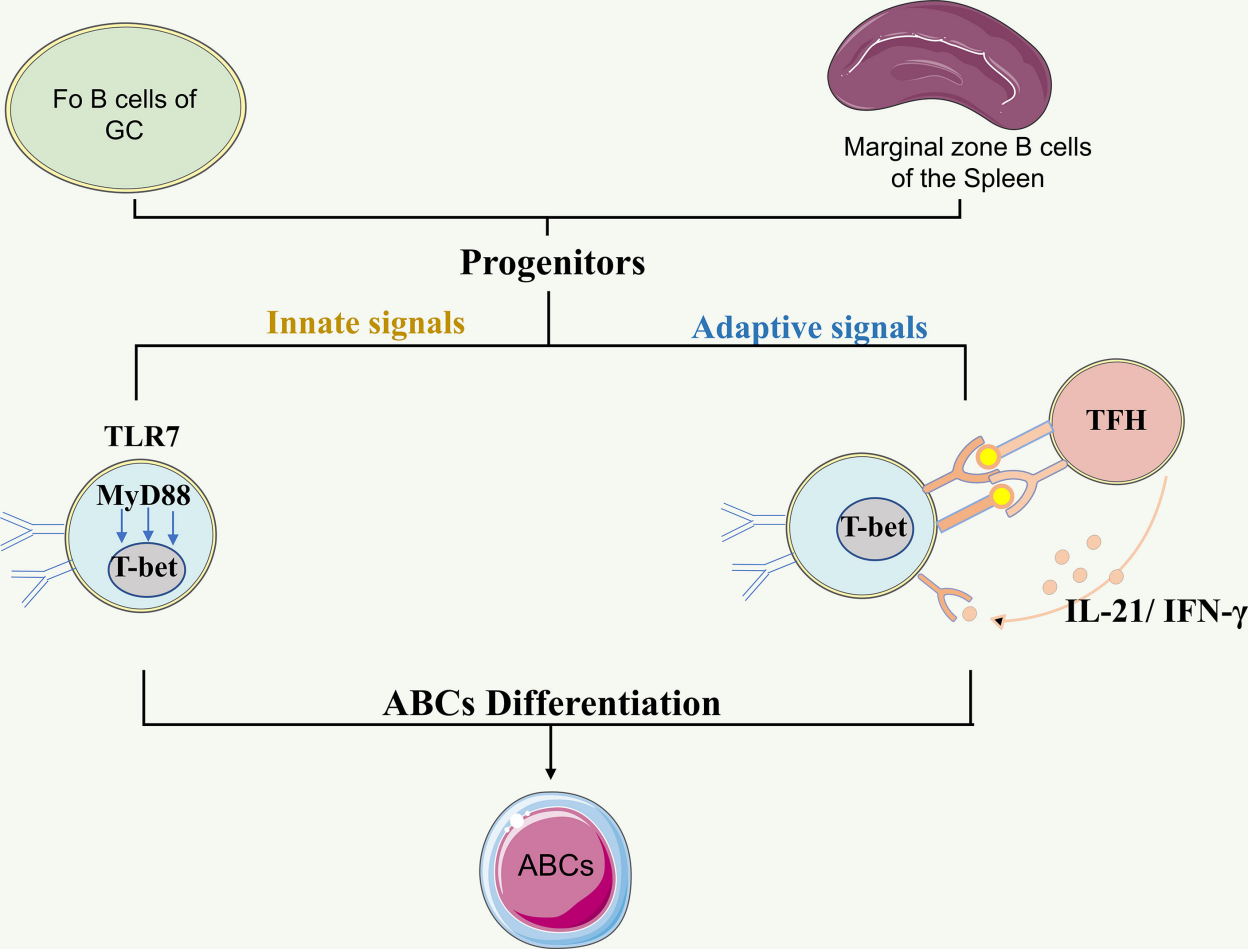
The origin and differentiation of ABCs. B cells from follicular and marginal zone of the spleen may serve as progenitors for ABCs. The differentiation of ABCs can be regulated by a combination of innate signals and adaptive signals. The innate signals TLR7 engagement followed by subsequent IFNγ and/or IL-21 exposure in the context of Tfh cells is required for ABC differentiation. The Figure was partly generated using Servier Medical Art, provided by Servier, licensed under a Creative Commons Attribution 3.0 unported license. ABCs: Age/autoimmunity-associated B cells; GC: germinal center; TLR7: Toll-like receptor 7; TLR9: Toll-like receptor 9; IL-21: interleukin-21; IFN-γ: interferon-γ.

Recently, by studying patients with defined inborn errors of immunity, Keller et al. demonstrated the essential role of BCR and T cell-derived IL-21 in the *in vivo* expansion of ABCs (56). They further observed a significant correlation between ABCs and circulating T follicular helper (Tfh) and T peripheral helper (Tph) cells, suggesting potential sources of CD40L, IL-21 and IFN-γ in promoting ABCs expansion (56). This is consistent with a finding in juvenile idiopathic arthritis, which demonstrated the expansion of Tph cells in the joints of JIA patients and revealed a positive correlation of synovial Tph frequencies with ABCs in situ (53). Importantly, synovial Tph cells skewed B cell differentiation toward ABCs phenotype *in vitro* by provision of IL-21 and IFN-γ (53). Altogether, the ability of ABCs to integrate both innate and adaptive signals may enable them to reflect the inflammatory clues in their microenvironment, thus allowing them to be an indicator of disease activity (**Figure 1**).

## 4 ABCs in rheumatoid arthritis

### 4.1 ABCs are expanded in the circulation and inflammatory joints of RA patients

RA is a prototype of immune-mediated inflammatory disease, characterized by persistent synovitis with leukocyte infiltration. Aberrant B cell phenotype and function have been conventionally recognized as one of the main contributors in the immunopathology of RA. Previous studies in other autoimmune diseases have described that ABCs are increased in the peripheral blood of RA patients (15, 22). Until recently, more attention has been paid on ABCs involvement in RA. Actually, increased frequency of CD27^-^IgM^-^IgD^-^CD21^-^ ABC-like B cells was observed in patients with seropositive RA compared with healthy individuals (72). Our group recently found that ABCs were expanded both in collagen-induced arthritis mice and in the circulation of RA patients with more severe symptom (30).Similarly, another group also demonstrated that circulating ABCs were increased in RA patients with higher disease activity and decreased in those with good treatment responses (73).

By integrating single-cell transcriptomics and mass cytometry, Zhang et al. defined inflammatory cell states in the joints and revealed that ABCs were one of the 5 populations expanded in the synovial tissue of RA patients (29). In line with these findings, we found the frequency of ABCs in the SF was more than 10 times higher than that in the peripheral blood and confirmed their accumulation in the synovium of RA patients by immunofluorescence and flow cytometry (30), raising the possibility that these cells are recruited to the joints during ongoing inflammation. Actually, the inflammatory chemokine receptor CXCR3, which is usually absent on circulating B cells, has been observed to be upregulated on ABCs from both mice and humans (49, 74, 75). Altogether, these data suggest that circulating ABCs are capable of migrating into the inflammatory joints through chemotaxis and contribute to the progression of chronic synovitis. In addition, latent γHV68 infected Type II collagen-induced arthritis (CIA) mice showed higher clinical scores and changes in paw heights. And ABCs were increased and display an inflammatory phenotype in the spleen of latent γHV68 infected CIA mice compared to CIA mice. Furthermore, the knockout of ABCs can inhibit the exacerbation of CIA, implicating that latent gammaherpesvirus exacerbates arthritis through modification of ABCs (76). Hence, ABCs might act as a key mediator in RA pathogenesis.

### 4.2 Mechanisms of ABCs contribution to RA pathogenesis

#### 4.2.1 Secretion of autoantibodies and proinflammatory cytokines

ABCs can secrete autoreactive antibodies, which may play an important role in many autoimmune diseases. In sjogren’s syndrome, ABCs secrete autoreactive antibodies including double-stranded DNA, insulin, and lipopolysaccharide (24). Rubtsov et al. found that the depletion of ABCs can reduce the autoreactive antibodies in autoimmune-prone mice (77). Researchers have confirmed that ABCs can produce plenty of antichromatin IgG2a in autoimmune mice, while the depletion of ABCs reduces IgG and IgG2a production (21). In addition, the long non-coding RNA XIST maintains X-inactivation of immune genes in cells, and the deletion of the long non-coding RNA XIST contributed to the formation of ABCs (78). In a humanized lupus erythematosus mouse model, ABCs were recruited to the inflamed site with the participation of IL-21 and TLR7/9 signals, and then IgG2a, IgG2b and IgG3 were generated (79). In addition to the production of autoreactive antibodies, it has been reported that excessive ABCs compromise antigen-specific GC B-cell responses and antibody-affinity maturation in lupus mouse models (33).

Except the secretion of autoreactive antibodies, ABCs also secrete a variety of cytokines, including IL-4, IL-17, IL-10, IFN-γ and tumor necrosis factor-α (TNF-α), to regulate the immune system of the body (14, 80, 81). Actually, ABCs display a distinct cytokine profile from other B cells in autoimmune diseases. For example, in patients with Crohn’s disease, ABCs are abundant in the gut and express large amounts of IL-6, IFN-γ and IL-12 (32). In MS patients, increased frequencies of ABCs are found both in the blood and in the cerebrospinal fluid, where they contribute to inflammation by induction of T cell responses and production of TNF-α and IL-10 (25).

Compared with CD11c^-^ naive B cells, ABCs in RA patients display higher TNF-α, IL-17, IL-21 but not IFN-γ mRNA expression (30). The distinct cytokine expression profile of ABCs from other B cells enables them to be the most pro-inflammatory B cell subset. However, it appears that ABCs also produce IL-4 and regulatory cytokines like IL-10, which has been known to exert anti-inflammatory effect in arthritis (82, 83). More research is needed to determine how the ABCs-derived cytokines are changed during the different phases of inflammatory diseases.

#### 4.2.2 Antigen presentation and activation of T cells

A previous study has shown that bone marrow B cells from RA patients expressed higher levels of CD86 than their osteoarthritis counterparts, suggesting that B cells in RA have the potential to act as APCs (84). Actually, ABCs can present antigens effectively and potentiate Th17 polarization *in vitro (* 14). In aged as well as autoimmune-prone mice, ABCs are localized at the T/B cell border in the spleen, present antigens to T cells, both *in vitro* and *in vivo*, more efficiently than FoB cells, indicating their ability to interact with T cells and activate antigen-specific T cells (85).

There has been evidence that ABCs promote pathogenic T cell activation during autoimmune and inflammatory diseases. In Crohn’s disease, CD4^+^ T cells produce higher IFN-γ when cocultured with ABCs compared to cocultured with other B cells (32). Consistently, patients with higher ABCs also displayed increased Th1 infiltration into the gut compared to those with less ABCs (32). Similar results were found in SLE. Conditional deletion of T-bet from B cells in lupus mice leads to decreased activation of both T cells and B cells (86).

#### 4.2.3 Interplay between ABCs and FLS

Under chronic inflammation, B cells accumulate in the synovial membrane in a process analogous to that in the germinal center (87). Indeed, RA synoviocytes enhance the production of immunoglobulins by activated B cells and were potent to support the differentiation of B cells into plasma cells infiltrated in the synovium (88). It has been demonstrated that FLS, the key effector cells in RA synovium, facilitate the migration of B cells beneath the synoviocytes *via* a mechanism dependent on stromal cell-derived factor-1 (SDF-1) and vascular cell adhesion molecule 1 (VCAM-1) (89). In addition, C-X-C motif chemokine ligand 13 (CXCL13) and C-C motif chemokine ligand 20 (CCL20) also play crucial roles in the accumulation of B cells within the inflamed synovium (90). Recently, it was shown that TNF-α enhanced adhesion of B cells to FLS *via* the expression of VCAM-1, further supporting the interaction of B cells with FLS (91). However, whether FLS could recruit ABCs from circulation and maintain their survival in the synovium remains unclear and thus need further investigation.

On the other hand, B cells can induce the activation of synoviocytes *via* IL-36 (92), which enables binding to IL-36R and interleukin-1 receptor accessory protein (IL-1RAcP), a member of the Interleukin-1 receptor family, and activate the nuclear factor-κB (NF-κB) and mitogen-activated protein kinase (MAPK) pathways to produce pro-inflammatory cytokines (93). When cocultured with ABCs *in vitro*, FLS displayed elevated expression of ICAM-1 and VCAM-1, along with increased production of IL-6, matrix metallopeptidase (MMP)-1, MMP-3 and MMP-13, suggesting that ABCs shifted FLS to an aggressive phenotype (30). Notably, ABCs-conditioned medium (ABCs-CM) exerted similar effects as ABCs in coculture system, indicating that the activation of FLS induced by ABCs is cell-contact independent (30). Mechanistically, ABCs-derived TNF-α promotes the upregulation of interferon stimulated genes in FLS. Furthermore, blockage of ERK1/2 and JAK-STAT1 pathway significantly inhibited ABCs-induced FLS activation (30). However, cell contact-mediated ABCs-FLS crosstalk cannot be excluded and deserves further study.

**Figure 2:**
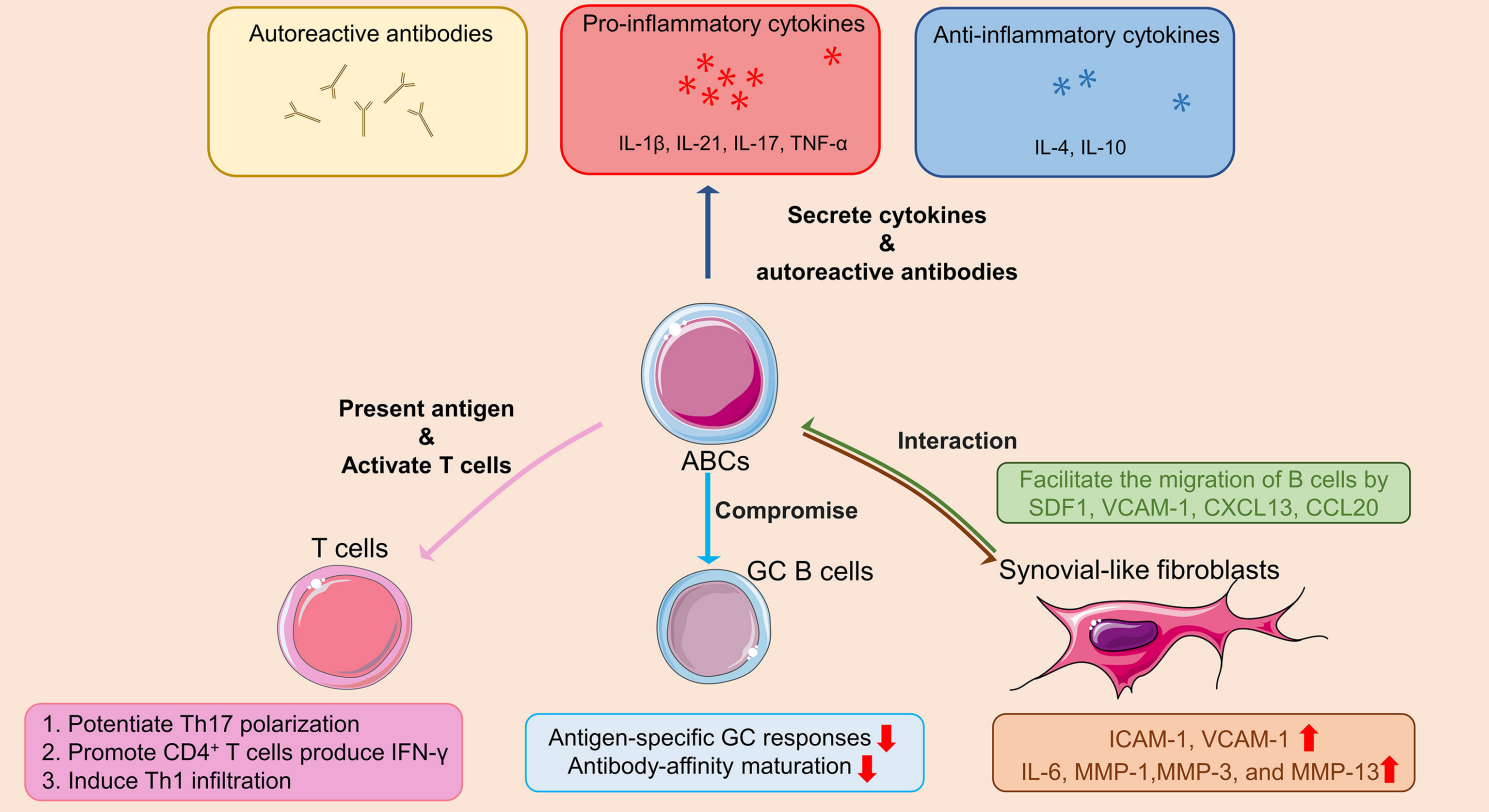
Schematic diagram outlining the role of ABCs in the pathogenesis of RA. In RA patients, ABCs secrete autoreactive antibodies to contribute to autoimmune diseases, inflammatory cytokines (i.e. TNF-α, IL-17 and IL-21) to promote inflammation, as well as anti-inflammatory cytokines (i.e. IL-4 and IL-10) with unknown effect. ABCs also act as antigen presenting cell promoting IFN-γ secretion of CD4^+^T cell, potentiating Th17 polarization, and Th1 infiltration. The antigen-specific GC responses and antibody-affinity maturation are compromised by ABCs in GC B cells. At sites of inflammatory arthritis, FLS secretes chemokines that facilitate the migration of ABCs into the inflamed joints, forming ABCs-FLS interaction and further inducing activation of FLS with increased inflammatory cytokines and metalloproteinases production. The Figure was partly generated using Servier Medical Art, provided by Servier, licensed under a Creative Commons Attribution 3.0 unported license. ABCs: Age/autoimmunity-associated B cells; RA: rheumatoid arthritis; TNF-α: tumor necrosis factor-α; IL-1β: interleukin-1β; IL-4: interleukin-4; IL-6: interleukin-6; IL-10: interleukin-10; IL-17: interleukin-17; IL-21: interleukin-21; IFN-γ: interferon-γ; GC: germinal center; FLS: synovial-like fibroblasts; SDF1: stromal cell-derived factor-1; VCAM-1: vascular cell adhesion molecule 1; CXCL13: C-X-C Motif Chemokine Ligand 13; CCL20: chemokine (C-C motif) ligand 20; ICAM-1: intercellular cell adhesion molecule-1; MMP-1: matrix metallopeptidase 1; MMP-3: matrix metallopeptidase 3; MMP-13: matrix metallopeptidase 13.

Taken above, the mechanisms by which ABCs contribute to the pathogenesis of RA are summarized in **Figure 2**.

## 5 ABCs in juvenile idiopathic arthritis

JIA is the most common rheumatic disease in children. It refers to a class of diseases related to chronic childhood arthritis, that begins before age 16 and persists for at least 6 weeks and cannot be attributed to any other cause (e.g. Lyme disease, septic arthritis). JIA not only affects joints, but also affects extra-articular structures, including eyes, skin, and internal organs, ultimately leading to disability and death without effective treatment. Although over the past decades, a variety of interventions have been developed to reduce JIA-induced tissue damage and eventually improve the quality of life of JIA patients, the complex pathogenesis of JIA remains incompletely understood (94, 95).

JIA is conventionally considered as a T cell-driven disease. However, the presence of anti-nuclear antibodies (ANAs) in almost half of the patients suggests that B cells are implicated in the pathogenesis of disease. Actually, many studies have shown that B cells play an important role in the occurrence and development of JIA through antigen presentation, cytokine secretion and autoantibody production (96–99).

Recently, one study explored the divergences of B cells in ANA^+^ JIA patients by assessing the distribution of B cell subpopulations in the peripheral blood and SF. They found no differences of B cell distribution in the peripheral blood between ANA^-^ and ANA^+^ patients. However, increased frequencies of CD21^lo/-^CD27^-^IgM^-^ DN2 B cells were observed in the SF of ANA^+^ JIA patients, suggesting that DN B cells are potentially involved in the development of disease and might be a characteristic subset expanding in the joints of ANA^+^ JIA patients (100). Another study investigated the phenotype and function of synovial CD4^+^ T cells that promote aberrant B cell activation in ANA^+^ JIA. Interestingly, the SF Tph cells can promote the differentiation of B cells toward the CD21^low/-^CD11c^+^ phenotype by providing IL-21 and IFN-γ. Additionally, SF Tph cell frequencies were positively correlated with SF CD21^low/-^CD11c^+^CD27^-^IgM^-^DN2 B cells *in situ* in JIA (53). Altogether, these findings suggest a model that, in arthritis settings, expanded Tph cells in the synovium might promote B cell differentiation into ABCs through the secretion of cytokines like IL-21 and IFN-γ.

## 6 ABCs-a potential therapeutic target

As the expansion and multiple functions of ABCs in diverse autoimmune diseases have been confirmed, the questions emerge: Whether the ABCs can be a potential therapeutic target? And if we can develop specific therapies based on ABCs for the treatment of autoimmune diseases?

Daniel Ramsköld et al. found that belimumab, a monoclonal antibody targeting the B cell cytokine BAFF, can decrease the ABCs with early clinical improvements in SLE in a prospective cohort study (101). Consistent with these findings, another clinical study showed that SLE patients who received rituximab treatment had a reduction of ABCs frequency at early follow-up (54). Besides, it was found that ABCs were reduced following plasma exchange treatment with a reduction in all immunoglobulin subsets in the circulation in patients with MS (102). More importantly, Gemma Vidal-Pedrola et al. found the ABCs express chemokine receptors and adhesion molecules that favor homing to inflammatory sites, which are the predominant B-cell subsets in SF at early RA (103). Taken together, these studies give us a hint that ABCs is a prognostic indicator in autoimmune diseases and acting as a potential therapeutic target. Notably, considering the increased risk of severe infections by depletion of total B cells, therapeutics that specifically target ABCs rather than pan-B cells would be a better strategy in the management of RA in the near future.

Some researchers have explored targeting ABCs for the treatment of autoimmune diseases. The adenosine receptor 2a (A2A receptor), also known as ADORA2A, is a potential target for immunotherapy. It is reported that the activation of A2A receptor can increase regulatory T cell generation, inhibit effector T cells and T follicular helper cells proliferation, and block the formation of GC B cells (104–107). Lymphocytes from RA patients are highly expressed A2A receptor, and the stimulation of A2A receptor can inhibit the production of pro-inflammatory cytokines *in vitro (* 108, 109). Moreover, the expression of A2A receptor is approximately 10 times higher than in CD11c^+^T-bet^+^B cells, compared to CD11c^-^B cells (110), suggesting A2A receptor may exert immunosuppressive effects by regulating ABCs. Indeed, Levack *el al.* depleted ABCs by the administration of the adenosine receptor 2a (ABCs-specific cellular marker) agonist CGS-21680, and they found that the depletion of ABCs reduced anti-nuclear antibodies in lupus-prone mice, accompanied by improved kidney pathology and lymphadenopathy (52). Although the reported effects of A2A receptor on autoimmune diseases are mainly focused on mouse ABCs, this targeted pharmacological approach for the elimination of ABCs also can be explored as a new drug in RA considering the highly expression of A2A receptor in RA lymphocytes. In addition, Sandra Hočevar et al. established polymer-coated gold nanoparticles, which can target ABCs and demonstrated the polymer-coated gold nanoparticles can serve as a safe tool to target ABCs *in vivo (* 111). And they confirmed that polymer-coated gold nanoparticles did not affect the percentages of other B cell populations in different organs. However, the development and application of treatments of polymer-coated gold nanoparticles has not been investigated. Therefore, targeted pharmacological therapy and nanomedicine based on ABCs will hopefully broaden the therapeutic prospects of ABCs in RA.

## 7 Future prospects

Considering the heavy burden of inflammatory arthritis on patients, new diagnostic and treatment methods for inflammatory arthritis are urgently needed. In the past decade, abnormal expansion of ABCs has been found to be associated with various autoimmune diseases. And researchers have made exciting progressions, including the discovery of the secretion of cytokines and autoantibodies by ABCs, the intercellular regulation of ABCs and other types of cells. It is precisely these features that make ABCs, a newly discovered cell subset in autoimmune diseases, as an important regulator of autoimmune responses and a potential therapeutic target.

Although it is clarified that the relationship of the ABCs and severity of arthritis, the precise mechanisms by which ABCs function during inflammatory arthritis requires additional delineation. Moreover, current studies have primarily examined ABCs separately in different immune settings in terms of autoimmune diseases. Wang et al. (22) performed a comparison of the CD11c^hi^ B transcriptional profile in individuals with healthy controls, SLE and RA. They found that the CD11c^hi^ B cells display a comparable transcriptional profile between SLE and RA. However, whether the ABC population displays related transcriptional profiles and exerts similar functions in inflammatory arthritis remains largely unexplored. Meanwhile, researchers have been investigating mouse and human ABCs separately. As shown in **Table 1**, different markers were used in the identification of mouse and human ABCs. However, ABCs with different markers may represent different stages of differentiation and perform different functions. Current research mainly focuses on the role of mouse ABCs in the development of various autoimmune diseases, but mouse models do not fully recapitulate human disease. Therefore, a systematic comparison of the commonalities and differences between mouse and human ABCs will promote the clinical translation of ABCs-related research. Furthermore, ABCs are a heterogeneous population, only approximately two-thirds of the cells are T-bet^+^, and among these, roughly half are CD11c^+^. It is unclear whether these cells represent stable, unrelated cell subpopulations, or whether they are at different stages of differentiation. Further studies to expand on the similarities and differences between ABCs in various immune settings will be important; Heterogeneity and differentiation characterization by single cell RNA sequencing would be valuable. With intense ongoing investigations in more areas, it will hopefully advance the understanding of the biology and function of ABCs in arthritis, anticipating novel insights into the pathogenesis of disease and targeting strategies of ABCs for the treatment of inflammatory arthritis.

## 8 Conclusions

A growing body of evidence has emerged in recent years that ABCs are implicated in the pathogenesis and development of inflammatory arthritis. However, knowledge of ABCs origin, differentiation, specific surface markers as well as their interactions with other immune cells are still limited, making it a great challenge to unravel the exact role of ABCs in the context of arthritis. More in-depth researches are needed for the better understanding of ABCs involvement in the different stages of arthritis. Thus, ABCs-based targeted therapies are anticipated for controlling inflammatory arthritis.

## Author contributions

Z-YL: Original draft preparation. M-LC: Original draft preparation. YQ: Conceptualization. ZC: Conceptualization, Supervision, and Funding acquisition. All authors contributed to the article and approved the submitted version.
